# Short-term clinical outcomes associated with local peloid therapy combined with exercise on pain, function, and quality of life in knee osteoarthritis

**DOI:** 10.3389/fphys.2026.1860407

**Published:** 2026-07-08

**Authors:** Nur Gökçe Aydın, Melek Bilgin, Halil İbrahim Kaya, Ahmet Serhat Genç, Burak Yoldaş, Samet Hasan Abacı, Enes Akdemir, Berna Anıl, Esra Korkmaz Salkılıç, Ali Kerim Yılmaz

**Affiliations:** 1 Department of Medical Ecology and Hydroclimatology, Samsun City Hospital, Samsun, Türkiye; 2 Department of Medical Microbiology, Samsun City Hospital, Samsun University, Samsun, Türkiye; 3 Department of Sports Medicine, Samsun City Hospital, Samsun, Türkiye; 4 Department of Orthopedics and Traumatology, Samsun City Hospital, Samsun, Türkiye; 5 Department of Orthopedics and Traumatology, Samsun Havza State Hospital, Samsun, Türkiye; 6 Department of Animal Science, Faculty of Agriculture, Ondokuz Mayıs University, Samsun, Türkiye; 7 Faculty of Yaşar Doğu Sports Sciences, Ondokuz Mayıs University, Samsun, Türkiye

**Keywords:** balneotherapy, exercise, knee osteoarthritis, peloid therapy, quality of life

## Abstract

**Background:**

To evaluate the short-term clinical and functional effects of local peloid therapy combined with exercise compared with exercise alone in patients with knee osteoarthritis (OA), using routinely collected clinical data.

**Methods:**

This retrospective comparative study included 70 patients with knee OA. Patients who received local knee peloid applications (30 minutes/day, 5 days/week for 3 weeks) combined with a standardized home exercise program (peloid + exercise; n = 35) were compared with patients who performed the same exercise program alone (exercise-only; n = 35) as part of routine clinical care. Outcome measures included pain [Visual Analogue Scale (VAS)], function [Western Ontario and McMaster Universities Osteoarthritis Index (WOMAC), Knee Injury and Osteoarthritis Outcome Score–Physical Function Short Form (KOOS-PS), Oxford Knee Score (OKS)], quality of life [Short Form-36 (SF-36)], functional performance [Five Times Sit-to-Stand Test (5xSTS)], range of motion (ROM), muscle strength, and selected systemic inflammatory markers [high-sensitivity C-reactive protein (hsCRP), tumor necrosis factor-alpha (TNF-α), Interleukin-6 (IL-6)].

**Results:**

Outcome data at week 3 were available for 60 participants (peloid + exercise n = 35; exercise-only n = 25). Both groups showed improvement in pain and functional outcomes, with more pronounced short-term improvements observed in the peloid + exercise group, particularly for pain scores, functional performance, muscle strength, and quality-of-life measures. No consistent between-group differences were observed in systemic inflammatory markers.

**Conclusions:**

In this retrospective analysis, the addition of local peloid therapy to exercise was associated with numerically and clinically more pronounced short-term clinical and functional improvements compared with exercise alone, while systemic inflammatory markers showed limited change. These findings support a potential complementary role for local peloid therapy in the short-term rehabilitation of knee OA.

## 1 Introduction

Osteoarthritis (OA) is a chronic, degenerative, and multifactorial joint disease that begins in the articular cartilage and leads to progressive damage to the subchondral bone and synovial structures over time (Hunter and Bierma-Zeinstra, 2019). This condition leads to a variety of serious clinical manifestations, such as pain, stiffness, swelling and limited joint function (Hawker, 2019). OA leads not only to loss of physical function but also to sleep disturbance, loss of the labor force, and decreased quality of life (Allen et al., 2022). OA constitutes a significant burden of disease and especially affects the quality of life of elderly individuals. Knee and hip OA cases are estimated to affect 300 million people worldwide, and this burden is expected to increase with the aging of the population (Safiri et al., 2020; Steinmetz et al., 2023). The knee joint is the most frequently affected site clinically; obesity is the major risk factor for knee OA, and the risk is increased approximately threefold in obese individuals (Jiang et al., 2012; Allen et al., 2022). The etiology of OA is multifactorial and includes inflammatory factors, metabolic factors, and mechanical factors (Jiang et al., 2012).

Mechanical stress, metabolic factors and inflammatory processes play critical roles in the pathogenesis of OA. Proinflammatory cytokines such as interleukin-1β (IL-1β), tumor necrosis factor-alpha (TNF-α) and interleukin-6 (IL-6) mediate the development of synovitis, cartilage destruction and disease progression (Mathiessen and Conaghan, 2017). High-sensitivity C-reactive protein (hsCRP) levels are considered potential biomarkers of synovitis but are not thought to have a direct effect on OA progression (Engström et al., 2009). The inflammatory process of OA is initiated in the synovial membrane by the activation of the immune system, which involves both humoral and cellular mediators (Millerand et al., 2019). Synovitis is a common finding in OA joints and is associated with clinical symptoms in patients (Sellam and Berenbaum, 2010). Therefore, therapies that reduce inflammation and restore function are essential in OA management. The interplay between the immune, nervous, and endocrine systems—referred to as the immuno-neuroendocrine axis—has also been suggested as a possible mechanism contributing to the effects of physical modalities such as peloid therapy (Fioravanti et al., 2017).

Current international guidelines recommend nonpharmacological approaches—particularly structured exercise programs—as the cornerstone of knee OA treatment (McAlindon et al., 2014; Arden et al., 2021). Exercise improves joint mobility, strengthens periarticular muscles, and reduces pain and disability. However, adherence to exercise alone is often limited, and combining exercise with complementary physical therapies may enhance clinical outcomes and patient satisfaction (McAlindon et al., 2014; Arden et al., 2021). Among conservative nonpharmacological modalities, local peloid therapy (therapeutic mud) has gained particular attention for its anti-inflammatory, analgesic, and functional benefits (Antonelli et al., 2018; Maccarone et al., 2021). While balneotherapy encompasses various spa-based thermal treatments, peloid therapy specifically involves the local application of mineral-rich mud directly to the affected joint area. In local peloid therapy, the peloid is typically applied in 1–2 cm thick layers, with application temperature varying depending on treatment protocols, thermophysical properties of the peloid, and clinical practice conditions. The thermophysical properties of peloids may support effective thermotherapeutic heat transfer at higher application temperatures, provided that the treatment remains well tolerated and does not produce adverse effects (Ferrand and Yvon, 1991; Maraver et al., 2021). Previous studies have reported that peloid therapy may be associated with reductions in cytokine levels such as TNF-α and IL-6 and contribute to clinical improvements (Fioravanti et al., 2017). Although the mechanism of local peloid therapy has not been fully explained, it is thought that mechanical, thermal and chemical effects combine to provide an potential anti-inflammatory effect (Bender et al., 2005; Fioravanti et al., 2011; Fioravanti et al., 2017). However, limited data are available regarding the simultaneous evaluation of both functional and inflammatory outcomes of short-term local peloid therapy when combined with exercise.

This retrospective comparative study aimed to evaluate the short-term clinical and functional effects of local peloid therapy combined with exercise compared with exercise alone in patients with knee OA, with additional assessment of selected systemic inflammatory markers. This study was designed to provide additional clinical data on the short-term effects of combining local peloid therapy with exercise and to inform future research on multimodal nonpharmacological management strategies for knee OA.

## 2 Materials and methods

### 2.1 Study design

**Figure 1:**
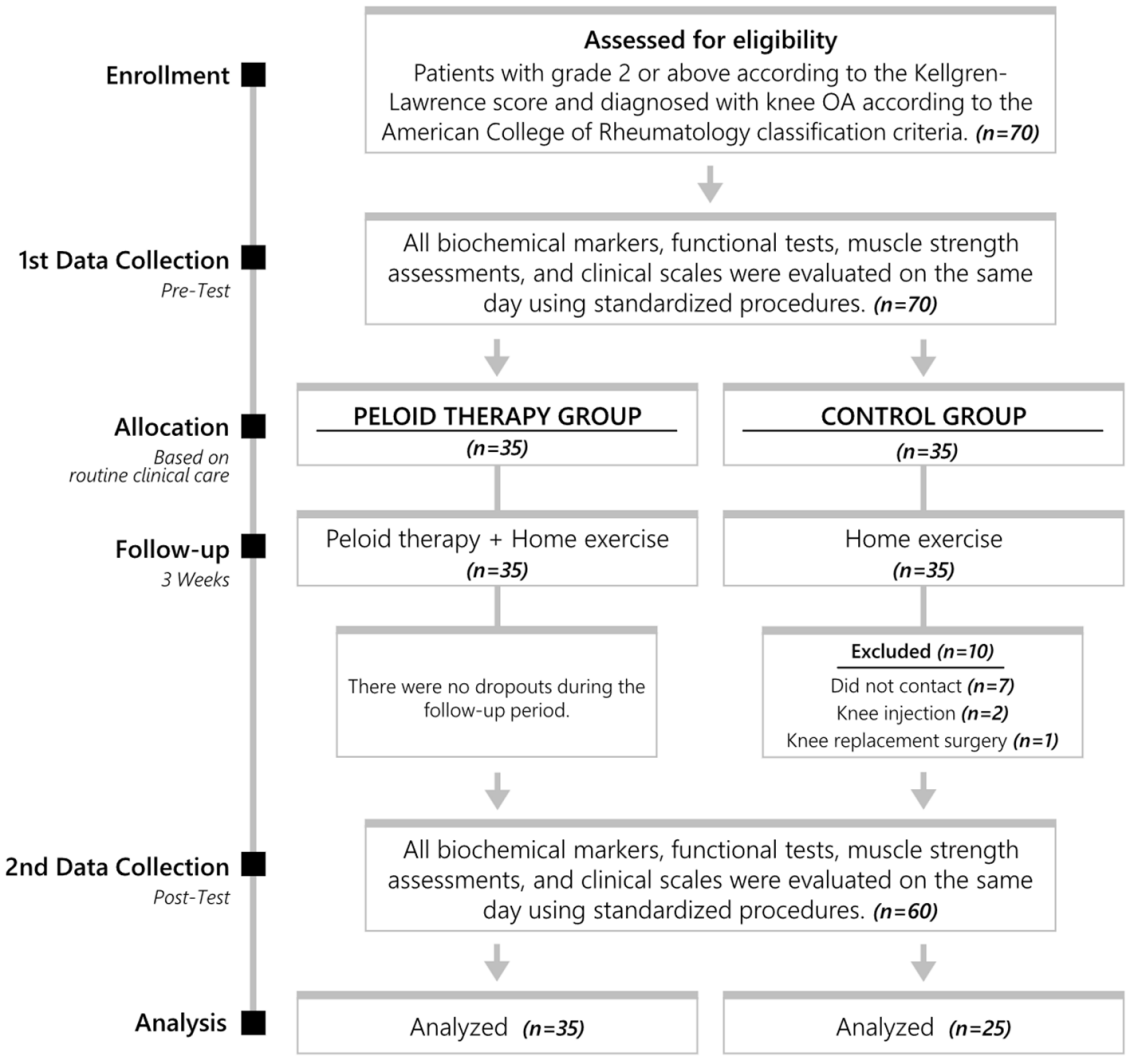
Flowchart.

This study was designed as a retrospective comparative cohort analysis based on routinely collected clinical data. Patients with knee OA who received local peloid therapy in addition to a standardized home-based exercise program were compared with patients who performed the same standardized exercise program alone during the same treatment period as part of routine clinical practice (**Figure 1**).

Ethical approval was obtained from the Samsun University Clinical Research Ethics Committee (Approval No: SÜKAEK/2023/16/22, dated 06 September 2023). All study procedures were conducted in accordance with the principles outlined in the Declaration of Helsinki. All participants provided written informed consent prior to enrollment.

### 2.2 Participants

Patients diagnosed with knee OA according to the American College of Rheumatology (ACR) classification criteria and with a radiographic grade of ≥2 on the Kellgren–Lawrence scale were eligible for inclusion. A total of 70 patients with knee OA were included and grouped according to the treatment received in routine care: 35 received peloid therapy combined with a home-based exercise program (peloid + exercise group), and 35 received exercise only (control group). During follow-up, all participants in the peloid + exercise group completed the study. In the control group, 10 participants did not complete follow-up due to loss to follow-up (n = 7) or initiation of additional clinical interventions (intra-articular injection, n = 2; knee replacement surgery, n = 1). As a result, outcome data at follow-up were available for 60 of the 70 included participants (peloid + exercise, n = 35; control, n = 25), and analyses were performed on participants with available follow-up data.

The exclusion criteria were as follows: (i) Significant pathologies of the lumbar spine, hip, or ankle that could affect study outcomes, (ii) Decompensated organ failure, (iii) Active malignancy, (iv) Hemorrhagic disorders, (v) Febrile infectious disease, (vi) Severe knee trauma or surgery within the past 6 months or intra-articular corticosteroid or hyaluronic acid injection during this period, (vii) Previous balneotherapy or peloid therapy within the last year.

As this was a retrospective analysis, no *a priori* sample size calculation was performed. The sample size was determined by the number of eligible patients available in the clinical records during the study period. However, a *post-hoc* power analysis was conducted to determine the statistical strength of the study. With a final sample size of 60 patients, the study achieved a statistical power of 80% to detect a medium-to-large effect size (Cohen’s d = 0.74) at a significance level α of 0.05.

### 2.3 Interventions

Patients in the peloid therapy group received local knee peloid applications at approximately 46–48 °C (30 minutes per session, 5 days per week for 3 weeks; total of 15 sessions) in addition to a standardized home exercise program. The control group participated in the same standardized home exercise program without receiving peloid therapy. Exercise instructions were standardized and provided to both groups to minimize performance-related differences. In the peloid therapy group, peloid was applied locally to both knees as a 1.5–2 cm thick pack. Following application, the peloid was wrapped with plastic film and covered with a thick cloth to preserve heat. The peloid used in this study was obtained from the Afyonkarahisar Sandıklı region and combined with thermal water from the same area. It is rich in organic components, including humic acid, humin, and lignin. The chemical composition of the peloid was analysed by the Afyonkarahisar Public Health Laboratory (**Table 1**). Participants were permitted to take paracetamol (≤2 g/day) if needed for pain relief. Use of paracetamol was recorded during follow-up and considered in the interpretation of pain-related outcomes. No adverse events, including skin burns, erythema, blistering, or other local or systemic reactions related to the 48 °C peloid application, were observed during the intervention period.

**Table 1:** Physicochemical properties of peloids.

Analyzed parameter	Unit	Method	Detection limit	Results
pH	pH Unit	Electrometric	0-14	8.74
Large Particles		Physical		None
Color		Physical		Light Brown
Odor		Physical		Normal
Consistency		Physical		Dry
Homogeneity		Physical		Homogenous
Solutions		Physical		None
Water Retention Capacity	% (w/w)	Gravimetric		78.3
Cellulose	g/L	Gravimetric		7.78
Total Organic Matter	% (w/w)	Gravimetric		11.63
Mineral Content	% (w/w)	Gravimetric		88.37
Lignin	g/L	Gravimetric		61.03
Total Inorganic Matter	% (w/w)	Gravimetric		88.37
Humic Acid	g/L	Gravimetric		22.35
Humin	g/L	Gravimetric		61.03
Hemicellulose	g/L	Gravimetric		7.78
Bulk Density (Volume Weight)	g/L	Gravimetric		1327.6
Soluble Carbohydrates	g/L	Gravimetric		8.64
Water (Moisture) Content (105 °C)	% (w/w)	Gravimetric		3.99
Hydrogen Sulfide	g/L	Titrimetric		0.011

#### 2.3.1 Exercise program

A sports medicine specialist instructed all participants in a home-based exercise program designed for knee OA. The program targeted the quadriceps, hamstrings, and hip muscles, emphasizing range of motion and flexibility. Exercises were demonstrated face-to-face, and participants received a written guide. Each exercise was performed 10 repetitions, twice daily, for 3 weeks, with minor adjustments based on pain or functional tolerance.

### 2.4 Outcome measures

Participants were evaluated at two time points as part of routine clinical assessment: before treatment (T0) and at the end of treatment (week 3, T1). Outcome assessments were performed by the same sports medicine specialist. Outcome measures were selected to capture the multidimensional effects of the interventions on pain, physical function, physical performance, muscle strength, quality of life, and inflammatory status in patients with knee OA. Patient-reported outcomes included pain and functional assessments using the Visual Analogue Scale (VAS), Western Ontario and McMaster Universities Osteoarthritis Index (WOMAC), Knee Injury and Osteoarthritis Outcome Score–Physical Function Short Form (KOOS-PS), Oxford Knee Score (OKS), and quality of life using the Short Form-36 (SF-36). Objective functional outcomes included the Five Times Sit-to-Stand Test (5xSTS), active and passive knee range of motion (ROM) measurements, and isometric muscle strength assessments for knee flexion and extension at 0° and 90° using an isometric dynamometer. Serum inflammatory markers, including hsCRP, TNF-$\alpha$ and IL-6, were evaluated to assess potential systemic inflammatory changes associated with local peloid therapy. These parameters were chosen to provide a comprehensive evaluation of short-term clinical, functional, and biological responses to the interventions.

#### 2.4.1 Pain, function, and quality of life assessments

VAS was used to assess the pain level as well as the overall clinical assessment of the patient and physician. A lower score in the measurement system indicates both a lower pain level and a more favourable general health status (Price et al., 1983).

The quality of life of the patients was evaluated with the SF-36 questionnaire. This scale examines quality of life in eight subdimensions: physical functioning, role limitations due to physical problems, bodily pain, general health perception, energy/vital vitality, social functioning, role limitations due to emotional problems and mental health. SF-36 scores range from 0 to 100, with higher scores reflecting better health status. Turkish validity and reliability study was conducted by Koçyiğit et al. (1999).

The WOMAC scale, which is widely used in the evaluation of OA-related symptoms, consists of three subscales: pain, stiffness and physical function. The total score reflects the severity of the disease. Higher scores indicate more severe symptoms and lower quality of life. Turkish validity and reliability study was conducted by Tüzün et al. (2005).

KOOS-PS is the short form of the KOOS, focusing on physical function limitations in daily living and physical activities. In the original scoring system, lower scores indicate better physical function and fewer limitations. In the present study, KOOS-PS scores were interpreted consistently with this direction, with decreases over time reflecting functional improvement (Paker et al., 2007).

The OKS is a 12-item questionnaire assessing knee function and pain. Its Turkish validity was established by Tuğay et al. (2016). In the present study, data were analyzed using the original scoring system developed by Dawson et al. (1998). Each item is evaluated on a 1–5 scale, yielding a total score between 12 and 60. Accordingly, lower total OKS scores strictly indicate better knee function and less pain.

#### 2.4.2 Inflammatory markers assessments

A total of 5 mL of venous blood was drawn from each patient and centrifuged at 4000 rpm for 5 minutes. Serum samples were stored at −80 °C until analysis. Serum hsCRP levels (normal range < 5 mg/L) were measured using a BN II nephelometer and its dedicated high-sensitivity CRP reagent (Siemens Healthineers, Erlangen, Germany). The detection range for the hsCRP assay was 0.1–50 mg/L, with a lower limit of detection (sensitivity) of 0.10 mg/L. Serum IL-6 and TNF-α concentrations were measured using commercially available enzyme-linked immunosorbent assay (ELISA) kits (FineTest, Hubei, China), according to the manufacturer’s instructions. Optical density was read at 450 nm using a microplate reader (Tecan Infinite M200 PRO, Austria), and cytokine concentrations were calculated from standard curves generated with recombinant standards. The detection ranges were 4.688–300 pg/mL for IL-6 (sensitivity 2.813 pg/mL) and 15.625–1000 pg/mL for TNF-α (sensitivity 9.375 pg/mL).

### 2.5 Statistical analysis

Descriptive statistics are presented as the mean ± standard deviation for normally distributed continuous variables, the median (interquartile range) for non-normally distributed variables, and frequency (percentage) for categorical data. The normality of continuous variables was assessed using the Shapiro-Wilk test. For normally distributed parameters, a two-way mixed-design analysis of variance (ANOVA) was conducted with “Group” (peloid therapy combined with exercise vs. exercise alone) as the between-subjects factor and “Time” (pre-treatment vs. post-treatment) as the within-subjects factor. Primary inferences were based on the Group × Time interaction, with effect sizes reported as partial eta squared (η²p). For significant interactions, *post hoc* pairwise comparisons were performed using Bonferroni correction. For variables violating the normality assumption, non-parametric tests were used. Within-group pre-to-post changes were analyzed using the Wilcoxon signed-rank test. Between-group treatment effects were evaluated by comparing the pre-to-post change scores (Δ) using the Mann-Whitney U test. Furthermore, to ensure the robustness of the findings against the baseline age imbalance, an age-adjusted ANCOVA was conducted as a sensitivity analysis for the primary outcomes. To preserve the clarity of the main data tables, the outcomes of this age-adjusted analysis are summarized textually in the Results section. The statistical significance level was set at p < 0.05. All analyses were performed using SPSS Statistics version 27.0 (IBM Corp., Armonk, NY, USA).

## 3 Results

At baseline, demographic and anthropometric characteristics were analyzed in participants with complete follow-up data (n = 60). However, a clinically relevant numerical imbalance in age was present, with a mean age of 60.66 ± 10.49 years in the peloid group and 51.96 ± 7.50 years in the control group. The mean body mass index (BMI) was 31.25 ± 4.66 kg/m² overall, and both groups were within the obesity range. Female participants predominated (83.3% of all participants), and the majority were married (88.3%) and retired or unemployed (83.1%). Although several baseline characteristics were broadly similar between groups, the clinically relevant age imbalance and differential attrition indicate that between-group comparisons should be interpreted cautiously (**Table 2**).

**Table 2:** Baseline demographic and clinical characteristics of the peloid therapy and control groups.

Variable	Total (n = 60)	Peloid therapy group (n = 35)	Control group (n = 25)
Age (years)	57.03 ± 10.24	60.66 ± 10.49	51.96 ± 7.50
Weight (kg)	80.24 ± 13.56	81.23 ± 11.46	78.79 ± 16.30
Height (cm)	160.20 ± 8.56	162.09 ± 6.97	157.46 ± 9.98
Body mass index (BMI, kg/m²)	31.25 ± 4.66	30.97 ± 4.61	31.66 ± 4.79
Waist circumference (cm)	100.84 ± 11.60	105.24 ± 9.69	94.05 ± 11.18
Hip circumference (cm)	113.09 ± 10.54	116.74 ± 9.72	107.45 ± 9.38
Waist-to-hip ratio (WHR)	0.89 ± 0.06	0.90 ± 0.06	0.87 ± 0.06
Sex, n (%)			
Male	10 (16.7)	7 (11.7)	3 (5.0)
Female	50 (83.3)	28 (46.7)	22 (36.7)
Marital status, n (%)			
Single	7 (11.7)	4 (6.7)	3 (5.0)
Married	53 (88.3)	31 (51.7)	22 (36.7)
Employment status, n (%)			
Not working	42 (71.2)	23 (39.0)	19 (32.2)
Working	10 (16.9)	5 (8.5)	5 (8.5)
Retired	7 (11.9)	7 (11.9)	0 (0.0)
Smoking status, n (%)			
Nonsmoker	48 (82.8)	31 (53.4)	17 (29.3)
Smoker	10 (17.2)	4 (6.9)	6 (10.3)

No statistically significant group × time interactions were observed for knee ROM parameters (p > 0.05), indicating similar temporal patterns between groups. In the control group, a small within-group reduction was observed in active left knee flexion (from 119.2 ± 9.85° to 114.0 ± 9.03°, p = 0.028); however, this change was not accompanied by a significant between-group difference or interaction effect. No statistically significant differences were observed between the groups regarding hsCRP, TNF-α and IL-6 parameters (p > 0.05 for all; r = 0.033, 0.096, 0.142 respectively). (**Table 3**).

**Table 3:** Changes in inflammatory markers and range of motion in the peloid therapy and control groups.

Variable	Group	Pre mean ± SD	Post mean ± SD	p^t^	ANOVA (p)			
					Group	Time	Group × time	η²p
ROM flexion active right (°)	Peloid therapy	114.7 ± 12.77	115.4 ± 14.08	0.765	0.565	0.516	0.282	0.020
	Control	118.0 ± 10.21	115.2 ± 7.61	0.177				
ROM flexion passive right (°)	Peloid therapy	132.3 ± 13.88	134.3 ± 14.56	0.209	0.862	0.112	0.987	<0.001
	Control	132.9 ± 12.32	134.9 ± 10.10	0.318				
ROM flexion active left (°)	Peloid therapy	114.7 ± 14.52	114.6 ± 10.47	0.937	0.452	0.064	0.079	0.052
	Control	119.2 ± 9.85	114.0 ± 9.03	0.028*				
ROM flexion passive left (°)	Peloid therapy	133.1 ± 17.69	132.2 ± 9.89	0.674	0.785	0.735	0.371	0.014
	Control	132.4 ± 13.28	134.5 ± 10.01	0.406				
Variable	Group	Pre median (IQR)	Post median (IQR)	p^w^	Δ (Post-pre) median (IQR)		Mann-whitney U	
							p	r
hsCRP (mg/L)	Peloid therapy	3.00 (3.94)	3.00 (2.87)	0.751	0.00 (0.82)		0.828	0.033
	Control	3.31 (2.84)	3.31 (2.07)	0.222	0.00 (0.72)			
TNF-α (pg/mL)	Peloid therapy	15.00 (223.00)	15.00 (119.00)	0.069	0.00 (11.50)		0.496	0.096
	Control	15.00 (70.00)	15.00 (50.00)	0.692	0.00 (70.00)			
IL-6 (pg/mL)	Peloid therapy	4.00 (8.09)	4.00 (4.44)	0.451	0.00 (0.00)		0.272	0.142
	Control	4.00 (0.00)	4.00 (0.00)	0.142	0.00 (0.00)			

*p<0.05 (Statistically significant values are also highlighted in bold.); SD, Standard deviation; IQR, Interquartile range; p^t^, Results of the paired sample t test; p^w^, Results of the wilcoxon signed-rank test; η²p, Partial eta squared for the Group × Time interaction; r, Rank-biserial correlation coefficient (effect size); hsCRP, High-sensitivity C-reactive Protein; TNF-α, Tumor Necrosis Factor-Alpha; IL-6, Interleukin-6; ROM, Range of Motion.

In the peloid therapy group, post-test results were significantly higher for all parameters except right 90° knee extension strength (p < 0.05). The main effect of time was significant for right 90° extension, left 90° extension and flexion strengths (p < 0.05). There was a significant group × time interaction in left 90°flexion strength (p = 0.006). There were significant differences between groups in right 0°flexion, left 0° extension, and flexion strengths (p < 0.05) (**Table 4**).

**Table 4:** Changes in muscle strength parameters in the peloid therapy and control groups.

Variable	Group	Pre mean ± SD	Post mean ± SD	p^t^	ANOVA (p)			
					Group	Time	Group × time	η²p
Right 90° extension	Peloid therapy	239.94 ± 81.30	257.60 ± 78.93	0.127	0.577	0.018*	0.817	0.001
	Control	249.16 ± 76.46	270.56 ± 89.61	0.054				
Left 90° extension	Peloid therapy	236.63 ± 89.06	264.77 ± 81.58	0.009*	0.537	0.002*	0.650	0.004
	Control	253.28 ± 82.09	274.64 ± 92.46	0.049*				
Left 90°flexion	Peloid therapy	137.54 ± 42.35^b^	160.83 ± 44.36^a^	<0.001*	0.999	0.005*	0.006*	0.125
	Control	149.12 ± 50.63^ab^	149.24 ± 46.56^ab^	0.987				
Variable	Group	Pre median (IQR)	Post median (IQR)	p^w^	Δ (Post-pre) median (IQR)		Mann-whitney U	
							p	r
Right 0° extension	Peloid therapy	142.0 (51.00)	150.0 (35.50)	0.001*	24.00 (41.00)		0.117	0.240
	Control	136.0 (26.00)	150.0 (31.00)	0.432	7.00 (35.00)			
Right 0°flexion	Peloid therapy	113.0 (43.00)	135.0 (38.50)	<0.001*	20.00 (37.00)		0.006*	0.417
	Control	119.0 (41.00)	130.0 (40.00)	0.407	5.00 (21.00)			
Left 0° extension	Peloid therapy	140.0 (44.00)	153.0 (30.00)	0.001*	11.00 (34.50)		0.024*	0.346
	Control	145.0 (21.00)	150.0 (29.00)	0.976	-1.00 (21.00)			
Left 0°flexion	Peloid therapy	111.0 (51.50)	127.0 (43.00)	0.001*	23.00 (36.50)		0.010*	0.397
	Control	122.0 (51.00)	132.0 (36.00)	1.000	1.00 (43.00)			
Right 90°flexion	Peloid therapy	136.0 (69.00)	153.0 (58.00)	0.001*	27.00 (43.50)		0.056	0.293
	Control	120.0 (37.00)	141.0 (51.00)	0.319	1.00 (52.00)			

*p<0.05 (Statistically significant values are also highlighted in bold.); SD, Standard deviation; IQR, Interquartile range; p^t^, Results of the paired sample t test; p^w^, Results of the wilcoxon signed-rank test; η²p, Partial eta squared for the Group × Time interaction; r, Rank-biserial correlation coefficient (effect size); ^ab^, Values shown with different letters indicate statistically significant differences according to Bonferroni *post hoc* tests; N·m, Newton-meter.

Both groups demonstrated within-group improvements over time in the 5xSTS test and VAS pain scores (p < 0.05). Across pain, functional, and quality-of-life measures, significant main time effects were consistently observed (p < 0.05). In the peloid therapy group, post-test scores were significantly higher for all parameters except the SF-36 General Health (p < 0.05). For all parameters except the SF-36 Vitality and General Health, group × time interactions and between-group differences were statistically significant (p < 0.05), indicating more pronounced short-term improvements in the peloid therapy group (**Table 5**).

**Table 5:** Changes in functional tests, pain scores, and quality of life measures in the peloid therapy and control groups.

Variable	Group	Pre mean ± SD	Post mean ± SD	p^t^	ANOVA (p)			
					Group	Time	Group × time	η²p
VAS Patient Global Assessment	Peloid therapy	71.43 ± 25.48^a^	42.00 ± 25.53^b^	<0.001*	0.135	<0.001*	<0.001*	0.283
	Control	53.60 ± 14.9^b^	44.00 ± 12.58^b^	<0.001*				
VAS Physician Global Assessment	Peloid therapy	71.14 ± 25.32^a^	41.29 ± 25.65^c^	<0.001*	0.187	<0.001*	<0.001*	0.327
	Control	53.60 ± 11.04^b^	45.20 ± 12.62^bc^	<0.001*				
VAS Pain	Peloid therapy	71.71 ± 23.98^a^	41.14 ± 26.54^c^	<0.001*	0.316	<0.001*	<0.001*	0.259
	Control	56.00 ± 11.99^b^	46.80 ± 13.45^bc^	0.001*				
SF-36 Physical Functioning	Peloid therapy	34.43 ± 21.95^b^	50.29 ± 21.62^a^	<0.001*	0.607	0.022*	0.001*	0.169
	Control	41.40 ± 21.14^ab^	38.40 ± 18.01^b^	0.336				
SF-36 Vitality	Peloid therapy	39.29 ± 22.17	49.57 ± 20.09	0.004*	0.787	<0.001*	0.713	0.002
	Control	41.40 ± 17.59	50.00 ± 18.48	0.004*				
SF-36 Mental Health	Peloid therapy	48.57 ± 21.28^b^	59.49 ± 21.03^a^	0.001*	0.173	0.003*	0.035*	0.074
	Control	59.40 ± 17.18^a^	61.32 ± 15.00^a^	0.409				
SF-36 General Health	Peloid therapy	44.86 ± 21.81	49.14 ± 21.16	0.183	0.435	0.538	0.203	0.028
	Control	44.00 ± 17.2	42.50 ± 18.79	0.616				
WOMAC Pain	Peloid therapy	11.63 ± 3.90^a^	8.40 ± 4.33^b^	<0.001*	0.214	0.012*	0.002*	0.153
	Control	8.68 ± 4.39^b^	9.04 ± 3.69^b^	0.689				
WOMAC Physical Function	Peloid therapy	40.66 ± 15.87^a^	27.94 ± 13.86^b^	<0.001*	0.473	0.004*	<0.001*	0.231
	Control	35.48 ± 11.21^a^	37.60 ± 12.05^a^	0.242				
WOMAC Total	Peloid therapy	59.85 ± 22.33^a^	41.41 ± 19.92^b^	<0.001*	0.847	0.002*	<0.001*	0.208
	Control	50.72 ± 17.69^ab^	52.27 ± 16.81^a^	0.604				
KOOS-PS	Peloid therapy	18.11 ± 6.49^a^	14.40 ± 5.71^b^	0.001*	0.156	0.008*	0.039*	0.071
	Control	18.32 ± 5.34^a^	17.84 ± 4.49^a^	0.664				
OKS	Peloid therapy	36.60 ± 9.33^a^	31.65 ± 9.31^b^	0.002*	0.335	0.082	0.008*	0.116
	Control	35.64 ± 8.60^a^	36.72 ± 8.88^a^	0.494				
Variable	Group	Pre median (IQR)	Post median (IQR)	p^w^	Δ (Post-pre) median (IQR)		Mann-whitney U	
							p	r
5xSTS	Peloid therapy	11.11 (4.36)	9.62 (2.40)	<0.001*	-2.18 (2.60)		0.038*	0.318
	Control	9.56 (2.50)	8.30 (2.28)	0.001*	-1.13 (2.27)			
SF-36 Role-Physical	Peloid therapy	0.00 (25.00)	25.00 (75.00)	0.001*	0.00 (50.00)		0.013*	0.357
	Control	0.00 (50.00)	0.00 (25.00)	0.483	0.00 (45.00)			
SF-36 Role-Emotional	Peloid therapy	0.00 (33.30)	33.30 (66.70)	0.019*	0.00 (33.40)		0.018*	0.343
	Control	0.00 (66.70)	0.00 (33.30)	0.261	0.00 (33.30)			
SF-36 Social Functioning	Peloid therapy	37.50 (25.00)	62.50 (25.00)	0.001*	12.50 (31.25)		0.037*	0.314
	Control	50.00 (35.00)	62.50 (12.50)	0.722	0.00 (37.50)			
SF-36 Bodily Pain	Peloid therapy	35.00 (22.50)	45.00 (25.00)	0.001*	10.00 (22.50)		0.015*	0.368
	Control	22.50 (35.00)	30.00 (35.00)	0.779	0.00 (25.00)			
SF-36 Health Change	Peloid therapy	25.00 (25.00)	50.00 (25.00)	0.005*	0.00 (25.00)		0.004*	0.410
	Control	25.00 (25.00)	25.00 (0.00)	0.356	0.00 (25.00)			
WOMAC Stiffness	Peloid therapy	6.00 (5.50)	3.00 (3.00)	0.003*	-1.00 (5.00)		0.002*	0.464
	Control	5.00 (2.00)	4.00 (3.00)	0.095	1.00 (2.00)			

*p<0.05; SD, Standard deviation; IQR, Interquartile range; p^t^, Results of the paired sample t test; p^w^, Results of the wilcoxon signed-rank test; η²p, Partial eta squared for the Group × Time interaction; r, Rank-biserial correlation coefficient (effect size); ^abc^, Values shown with different letters indicate statistically significant differences according to Bonferroni post hoc tests; 5xSTS, Five Times Sit-to-Stand Test; VAS, Visual Analogue Scale; SF-36, Short Form-36; WOMAC, Western Ontario and McMaster Universities Osteoarthritis Index; KOOS-PS, Knee Injury and Osteoarthritis Outcome Score–Physical Function Short Form; OKS, Oxford Knee Score.

Although both groups showed improvement over the 3-week intervention period, the peloid + exercise group demonstrated a more consistent pattern of improvement across pain, functional performance, muscle strength, and quality-of-life domains. Taken together, these findings suggest a possible association between the addition of local peloid therapy and more pronounced short-term clinical and functional improvements, while acknowledging the short follow-up period and imbalanced attrition between groups.

An age-adjusted ANCOVA was performed to determine whether the baseline age imbalance confounded the treatment outcomes. The inclusion of age as a covariate did not alter the statistical significance levels of the primary parameters, confirming that the observed clinical improvements were robust and independent of baseline age differences.

## 4 Discussion

This study evaluated the short-term clinical and functional effects of local peloid therapy combined with exercise in patients with knee OA, as well as changes in selected inflammatory markers, using routinely collected clinical data. The findings were associated with improvements in functional capacity, muscle strength, quality of life, and pain scores in the peloid therapy group. In addition, for several muscle strength parameters and functional tests, significant group × time interactions and between-group differences were observed, indicating different short-term change patterns during routine clinical care. These findings suggest a possible association between the addition of local peloid therapy and short-term clinical and functional improvements in patients with knee OA.

A different trend was observed for the inflammatory parameters. Although the serum hsCRP, TNF-α, and IL-6 levels did not differ significantly between the groups, minor within-group differences were noted without clear clinical significance. These findings may suggest that short-term local peloid therapy is associated primarily with local or functional mechanisms rather than measurable systemic modulation of inflammatory markers. Therefore, the absence of significant changes in serum hsCRP, TNF-α, and IL-6 levels does not exclude localized biological effects at the joint level that may not be reflected by systemic measurements.

Previous studies indicate that the effects of balneological interventions on inflammatory responses may vary depending on treatment characteristics and the area of application (Alp et al., 2025). Since peloid therapy was applied locally to the knee region, it is plausible that no significant systemic reductions in inflammatory markers were observed. Moreover, both groups had mean BMI values within the obesity range (peloid group: 30.97 ± 4.61; control group: 31.66 ± 4.79), which may have maintained a state of low-grade systemic inflammation. These findings suggest that localized or functional mechanisms, rather than systemic inflammatory modulation, may underlie the observed clinical improvements and warrant further exploration. The relationship between obesity and knee OA has been well established, and this association is thought to be mediated by metabolic risk factors and the persistent low-grade inflammation often present in obese individuals (Pottie et al., 2006; Findlay, 2007; Rojas-Rodríguez et al., 2007). This metabolic background may have limited systemic cytokine modulation and contributed to unchanged hsCRP levels.

It has been reported that inflammatory processes in the synovium play important roles in the pathogenesis of OA and that the presence of inflammatory synovial infiltrates is associated with more severe symptoms, such as pain, swelling and joint dysfunction, in knee OA patients (Scanzello et al., 2011; Scanzello and Goldring, 2012). The levels of inflammatory mediators such as TNF-α and IL-6 play a role in the onset and severity of synovitis and OA symptoms (Sellam and Berenbaum, 2010; Berenbaum, 2013). High BMI and increased levels of circulating IL-6 are also associated with the development of radiologic knee OA (Livshits et al., 2009). These inflammatory mediators may represent potential therapeutic targets to relieve symptoms and prevent structural joint damage in OA (Scanzello and Goldring, 2012).

Previous studies evaluating whole-body pelotherapy have reported reductions in systemic inflammatory cytokines and proposed possible anti-inflammatory or chondroprotective mechanisms of mud therapy (Ortega et al., 2017). However, in our study, local application to only the knee region may be the main reason why no significant decrease in systemic inflammatory parameters was observed. Similarly, no significant change in TNF-α or IL-6 levels was found in studies in which mud was applied only to the knees (Sarsan et al., 2012). Similarly, in a study in which local peloid application was combined with hydrotherapy in patients with knee OA, no clinically significant improvement in IL-1β or insulin-like growth factor 1 (IGF-1) levels was found, and TNF-α levels could not be evaluated due to kit problems (Adıgüzel et al., 2022). These findings suggest that local peloid applications may have limited effects on inflammatory parameters and that investigating peloid applications covering larger body regions or combinations with other physical therapy modalities to obtain systemic responses may be useful.

The functional and clinical improvements observed in our study are consistent with previous studies reporting clinical improvements associated with balneological treatments in knee OA (Karagülle et al., 2007; Fioravanti et al., 2010; Forestier et al., 2010; Tefner et al., 2013; Şahin Onat et al., 2015; Hou et al., 2020; Varzaityte et al., 2020; Mennuni et al., 2021; Protano et al., 2023; Aydın et al., 2024). Peloid therapy has been shown to have beneficial effects on function, pain and quality of life in patients with OA and may represent a useful complementary option in clinical management (Espejo-Antúnez et al., 2013; Espejo-Antunez et al., 2013; Fioravanti et al., 2015). The thermal and mechanical effects of peloid therapy, combined with its capacity to promote muscle relaxation, enhance peripheral circulation around the joint, and act synergistically with exercise, are considered the main mechanisms underlying its potential clinical effects (Mennuni et al., 2021; Ortega-Collazos et al., 2024; Rossi, 2024; Shoubir et al., 2024). In addition, the ability of balneological treatments to reduce muscle spasms, increase ROM and improve functional mobility has been documented (Şahin Onat et al., 2015; Fraioli et al., 2018; Varzaityte et al., 2020; Ortega-Collazos et al., 2024). The observed improvements in several muscle strength and functional outcomes are broadly consistent with these mechanisms and are reflected in the positive changes in the SF-36 subparameters and WOMAC and KOOS-PS scores, indicating an overall improvement in quality of life.

The strengths of this study include the multidimensional assessment of knee OA using routinely collected clinical data, incorporating patient-reported outcomes, objective functional tests, muscle strength measurements, and inflammatory markers. The combined evaluation of local peloid therapy alongside a standardized home-based exercise program reflects real-world clinical practice and provides a pragmatic perspective on multimodal nonpharmacological management. Several limitations should also be acknowledged. The relatively short follow-up period and modest sample size limit the ability to draw conclusions regarding long-term outcomes and broader generalizability. In addition, the absence of consistent changes in systemic inflammatory markers, the clinically relevant age imbalance between groups, and the differential attrition during follow-up necessitate cautious interpretation of the findings. In particular, complete follow-up in the peloid group but fewer completers in the control group may have introduced selection bias and affected between-group comparisons. Consequently, an intention-to-treat (ITT) analysis could not be reliably performed due to the lack of post-treatment data for these dropouts, and efficacy outcomes were evaluated strictly on a per-protocol basis. Although paracetamol use was permitted and recorded during follow-up, detailed analyses regarding frequency, cumulative dosage, or between-group distribution were not performed. Therefore, the potential influence of analgesic use on pain- and function-related outcomes cannot be fully excluded. As both groups received a standardized exercise program known to improve symptoms in knee OA, the observed between-group differences should be interpreted as suggestive of a potential complementary benefit of local peloid therapy rather than definitive evidence of superiority. Accordingly, the findings should be regarded as exploratory and hypothesis-generating within the context of a retrospective comparative design, and should not be interpreted as establishing causal relationships. Despite these limitations, the consistency of improvements across multiple clinical and functional outcomes suggests that local peloid therapy may be associated with additional short-term benefits when integrated into routine exercise-based management of knee OA.

Future research with larger, multicenter trials and longer follow-up durations could help clarify the long-term systemic and local effects of peloid therapy, including the impact of the application area and treatment duration.

## 5 Conclusions

In this retrospective comparative study, short-term local peloid therapy combined with exercise was associated with improvements in functional capacity, muscle strength, pain, and quality of life in patients with knee OA. Although changes in systemic inflammatory markers were limited, the combined approach was associated with more pronounced short-term improvements across several outcome domains compared with exercise alone. Within the context of the study’s exploratory design and methodological limitations, local peloid therapy may represent a potential complementary nonpharmacological approach for short-term symptom management in knee OA.

## Data Availability

Data supporting the findings of this study are available through the corresponding author, but restrictions apply to the availability of these data used for the current study and are therefore not publicly available. However, data are available from the corresponding author upon reasonable request.
